# Increase in the Number of Military Veterinary Surgeons

**Published:** 1896-06

**Authors:** 


Increase in the Number of Military Veterinary Surgeons. In accordance with an order of his majesty, the Emperor, the state of the military veterinary classes in time of peace, from June 1, 1896, is as follows: 22 superior veterinary surgeons of the first class; 22 superior veterinary surgeons of the second class; 44 military veterinary surgeons; and 36 inferior military veterinary surgeons.—*Ibid.*

